# Rewilding’s social–ecological aims: Integrating coexistence into a rewilding continuum

**DOI:** 10.1007/s13280-024-02118-0

**Published:** 2024-12-31

**Authors:** Sally Hawkins, Steve Carver, Ian Convery

**Affiliations:** 1 Institute for Science and Environment, University of Cumbria, Rydal Road, Ambleside, UK; 2 School of Geography, University of Leeds, Leeds, UK

**Keywords:** Coexistence, Resilience, Rewilding, Social–ecological systems, Transformation

## Abstract

This paper presents results from a grounded theory study of rewilding aims, addressing calls for broad scale studies of rewilding to contribute to the development of guidelines. The grounded theory draws from a broad set of data sourced from rewilding organizations, case studies, and research. Expressions from the data relating to rewilding aims and outcomes were coded. The results demonstrate the intentions for rewilding to affect systemic, ecological, and socio-cultural change. Outcomes to support rewilding aims are also identified. The aims and outcomes are presented under these headings in a social–ecological framework which offers a shared vision for rewilding. The significance of this research is that it demonstrates rewilding’s multi-disciplinarity and engagement with systemic or transformative change. It addresses a perceived paradox between rewilding intervention and non-human autonomy, demonstrating that rewilding is not necessarily about removing human influence but affecting coexistence through more-than-human collaboration. A revised rewilding continuum integrating coexistence is proposed.

**Supplementary Information:**

The online version contains supplementary material available at 10.1007/s13280-024-02118-0.

## Introduction

While there has been ongoing work to better articulate rewilding’s aims and principles (e.g. Prior and Ward 2016; Carver et al. 2021; Hawkins 2022), rewilding remains a complex concept, and as such debate and uncertainty around its theoretical assumptions, practice, and potential remain. A key area of uncertainty and debate is around the role of people and culture in relation to wildness and non-human autonomy, linked to dualistic or holistic ontologies that perceive nature and culture as separate or integrated, respectively (Desilvey and Bartolini 2018; Gammon 2019; Holmes et al. 2020; Wynne-Jones et al. 2020a; Schulte to Bühne et al. 2021). These have influenced variation in rewilding aims, practices, and decision-making relating to how non-human autonomy is interpreted, e.g. whether rewilding aims for “pristine” wilderness, without human influence; aims to integrate wildness and culture in shared landscapes; or the extent to which rewilding aims to promote human flourishing and well-being (Deary and Warren 2019; Holmes et al. 2020; Wynne-Jones et al. 2020b). These aims are often perceived as conflicting, and this is reflected in the literature, prompting, for example, criticisms of the wilderness concept (e.g. Ward 2019) or criticisms of anthropocentrism in rewilding projects or theories (e.g. Genes et al. 2019; Kopnina et al. 2022), echoing similar debates in the wider conservation movement (Holmes et al. 2017; Sandbrook et al. 2019).

When viewed from a more holistic ontology, rewilding does not necessarily mean the total retreat of humans (Wynne-Jones et al. 2020a), rather it grants greater agency to non-human actors as co-creators of cultural landscapes (Gammon 2019). However, this is not easily reconcilable with existing rewilding frameworks as they are predicated on the notion of withdrawing human influence. For example, frameworks established by Perino et al. (2019), Torres et al. (2019), and Van Meerbeek et al. (2019) reflect that rewilding acts on a gradient from human intervention to ecological integrity, reflecting Carver’s (2014) adaptation of the wilderness continuum from Leslie and Maslen (1995), which suggests that rewilding occupies a specific window along a continuum of decreasing anthropogenic influence and increasing ecological integrity. These frameworks have led to a perceived paradox between rewilding interventions and the aims of non-human autonomy, and are highlighted as a barrier to rewilding application as rewilding continues to be perceived by some as the total withdrawal of human influence (Wynne-Jones et al. 2020b). In a study of rewilding practice and policy in Britain, Wynne-Jones et al. (2020b) demonstrate that rewilding projects are moving away from binaries that influence a more “hands-off” approach, offering a more relational approach to enhancing human–nature connectivity and livelihood opportunities. In this framing then, the perceived paradox between intervention and non-human autonomy is alleviated.

Adding to these uncertainties, there are some known limitations to existing rewilding theories in the academic literature: that there is a lack of empirical evidence informing rewilding theory, that academic literature reviews are limited given the prevalence of grey literature influencing rewilding conceptualisations, and that these limitations have fuelled debate and conflicting opinions (Johns 2019; Holmes et al. 2020; Fisher and Carver 2022). There are an increasing number of empirical studies that address these issues, but these tend to focus on geographical areas, e.g. Britain (Wynne-Jones et al. 2020b) or Europe (Holmes et al. 2020), or specific case studies (e.g. Desilvey and Bartolini 2018; Dempsey 2021), thereby limiting their applicability to international policy. There remains a need to better integrate varying perspectives and influences on rewilding aims and definitions, to clarify how rewilding interventions correspond with notions of other-than-human agency and justice, and to develop a rewilding framework that reflects the plurality of rewilding while demonstrating common ground.

This article addresses this by examining rewilding aims with the use of grounded theory, drawing on a combination of surveys providing personal accounts of those shaping rewilding theory and practice, along with texts (including academic and grey literature) considered influential to rewilding theory and practice. The study demonstrates that rewilding seeks to balance ecological aims of non-human autonomy with systemic and socio-cultural aims to support sustainable and equitable rewilding at scale. Building on this study of rewilding’s social–ecological aims, a revised rewilding continuum is proposed which reflects a key rewilding outcome of coexistence at landscape scale, addressing concerns with existing frameworks noted above.

## Materials and methods

Grounded theory (GT) is a form of exploratory research that is especially useful for clarifying and synthesizing existing theories and conceptualisations of complex concepts (Glaser and Strauss 1965; Stebbins 2001). It allows for flexible data collection and analysis, with the researcher exploring data for patterns, ideas, or hypotheses to produce inductively derived generalizations about the topic under study, weaving these into a “grounded theory” that goes some way to explaining the phenomenon as experienced by people operating within (Stebbins 2001; Creswell 2007; Charmaz 2014).

### Data collection

This study draws upon two primary data sources: the rewilding pioneer survey (RPS) and influential rewilding texts (IRT).

#### Rewilding pioneer survey (RPS)

The RPS was originally designed to support the work of the IUCN Commission for Ecosystem Management (CEM) Rewilding Thematic Group (RTG) in developing guiding principles for rewilding (Carver et al. 2021). It targeted individuals recognized as influential figures in the development of the rewilding field, referred to as “rewilding pioneers”, identified through rewilding publications (books and peer-reviewed journal articles), self-identification, and the snowball method, with the survey containing a question asking for recommendations. The survey encompassed 19 predominantly open-ended questions (see Supplementary Information) related to the participants’ conceptualisations and experiences of rewilding in the past and present, therefore considering how these have changed over time and influences on their conceptualisations and practice. It was conducted in 2018, yielding 60 responses (out of 126 invitations to participate). Participants represented diverse backgrounds, including academics, authors, and practitioners from various disciplines, with many associated with well-known rewilding organizations or widely cited rewilding publications. The participant composition leaned towards North America (27) and Western Europe (25), aligning with the survey’s focus on “pioneers” and the historical roots of rewilding in the USA and Western Europe since the 1980s. However, there was some representation from Australia (6), Africa (2), Asia (1), and South and Central America (2; note that where more than one country of residence was listed both were counted).

#### Secondary material: Influential rewilding texts (IRT)

Reflecting the iterative nature of constructivist grounded theory (Charmaz 2014), the second dataset consists of texts cited by RPS respondents as being influential to their conceptualisations and practice of rewilding, with many responses prompting further exploration of these texts. The IRT encompasses 10 journal articles, nine non-peer-reviewed articles (including policy briefs, magazine articles, and speeches), six single-author books, four edited books, and an additional book chapter. A comprehensive list can be found in Supplementary Table S1. Note that some texts are not about rewilding; however, they are considered as influential to the rewilding concept based on the comments from RPS respondents and were considered in this way, rather than as representing rewilding. Given the breadth of texts identified in the RPS, all texts cited in the RPS were used and this allowed us to delimit a clear set of influential texts among proliferating rewilding literature. This also allowed us to include influential texts that are not included in academic literature databases and are therefore not often included in existing rewilding literature reviews, which is considered by some as a shortfall in existing rewilding theories (Johns 2019; Fisher and Carver 2022). This selection of texts is believed to provide valuable insights from influential figures on the rewilding concept, address gaps in cases where influential figures had not participated in the RPS, and represent a range of influential rewilding organizations or projects.

### Data analysis

The data analysis process was conducted using NVivo 12. The RPS data and IRT material were uploaded where possible. Where IRT pdfs were not available, page references were inserted in the respective code in NVivo. RPS data coding was carried out during 2020, while IRT coding took place over 2021 and 2022. Based on an initial literature review, the RPS data were coded under three parent nodes focused on the concept of change: “change what” (intended impacts), “change why” (context and drivers), and “change how” (application or pathways). The IRT allowed for a deeper exploration of the themes emerging from the initial coding of the RPS data, leading to further refinement, analysis, and conceptualization of the initial codes. During this process, codes became more precisely defined, sub-nodes emerged, and certain categories were repositioned under different parent nodes, while the overarching parent nodes remained consistent. The coding process allowed the data to be viewed together under these themes, with the researcher able to see progression of these themes over time and links between them.

The three parent nodes correspond with the three main sections of a theory of change framework (Biggs et al. 2017; Ghate 2018; Reinholz and Andrews 2020), and the codes emerging under “change what” were found to reflect the aims/vision and outcomes of theory of change frameworks. This article primarily presents the findings related to the parent node “change what”, while the findings related to the other parent nodes are presented elsewhere (Hawkins 2023; Hawkins et al. 2024).

## Results

**Fig. 1:**
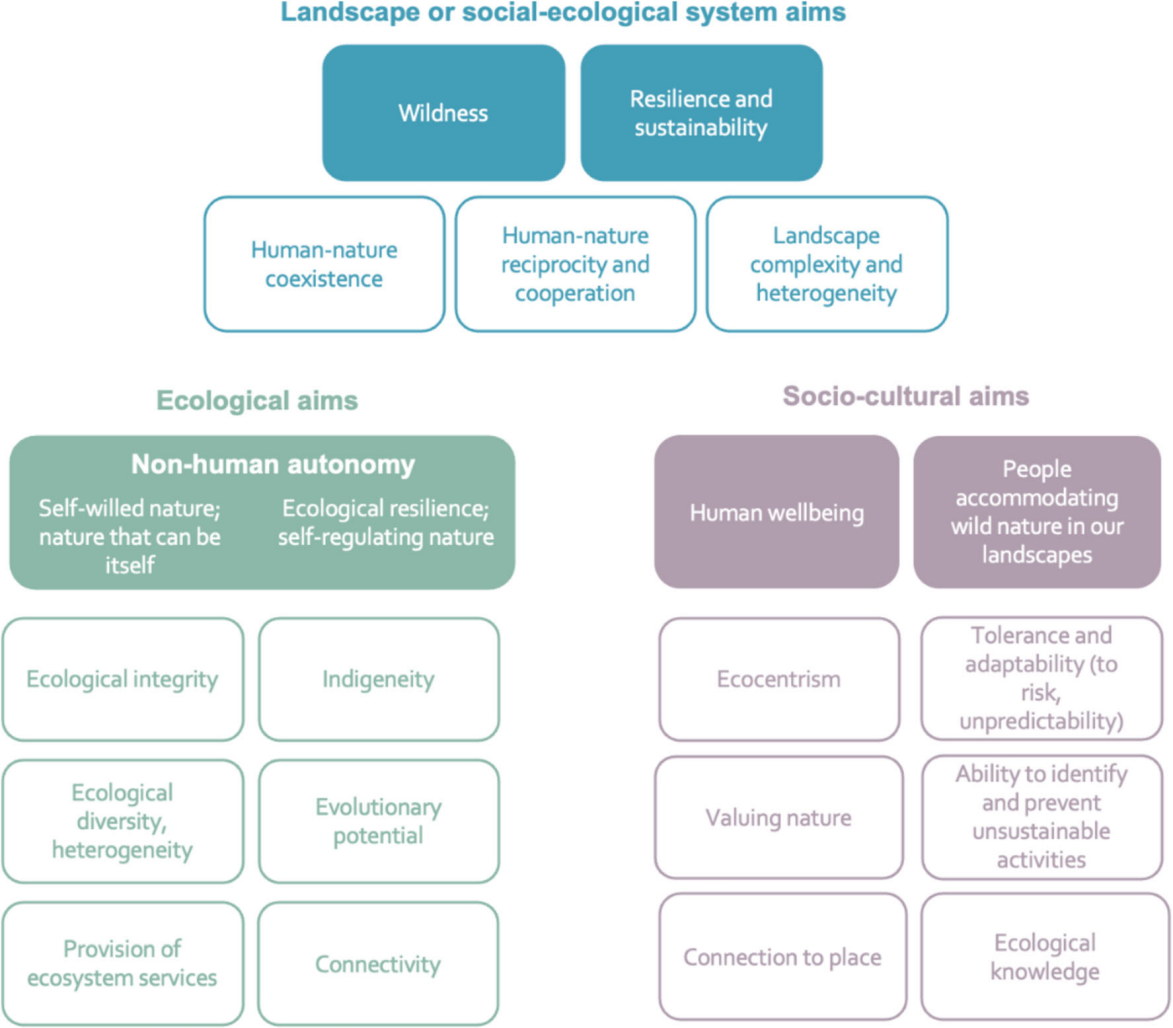
The social–ecological aims of rewilding, displaying the landscape scale or SES, ecological, and socio-cultural aims of rewilding together with outcomes that are identified as positively contributing to these aims. These are discussed separately in the relevant sections below. Filled boxes = rewilding aims; unfilled boxes = outcomes contributing to rewilding aims

The intended impacts of rewilding emerging from the data relate to change in three different categories—change to landscapes or social–ecological systems (SES), ecological change, and socio-cultural change. These formed three sub-nodes of the parent node “change what”. The themes emerging under these sub-nodes are presented as SES, ecological, and socio-cultural aims and outcomes for rewilding (Fig. 1). While there is some overlap with these themes, we have opted to consider them separately as they have varied implications for rewilding as noted below. This also aligns with constructivist grounded theory which deconstructs concepts to inform emergent theories (Charmaz 2014). Given limitations of space, we have focused descriptions mainly on the aims (filled in boxes in Fig. 1), while the outcomes are presented, and links are noted.

A continuum for rewilding based on coexistence is proposed in the concluding section. When seen together, the aims and outcomes presented here demonstrate the potential for rewilding to promote paradigm shifts and transformative change. The study establishes some general commonalities in the intentions of those engaged in rewilding theory and practice, although some variation in these are highlighted below. This study offers a holistic picture of rewilding aims, providing a focus for rewilding practice, further rewilding research, and the development of rewilding frameworks or metrics. The aims and outcomes presented here can be associated with the vision and outcomes of a theory of change framework, respectively, with the vision concerning intended long-term changes and the outcomes providing the preconditions that contribute to the vision, potentially serving as measurable indicators (Hawkins et al. 2024). Given limitations of space and time, future studies of these themes are warranted.

### SES or landscape-scale change

The data reflect systemic and landscape-scale change echoing the growing integration of systems thinking in rewilding theory and practice (Jones and Jones 2023; Hawkins et al. 2024). However, the lack of clarity and subjectivity around the aims of wildness, sustainability, and resilience are highlighted below. This study of rewilding aims and outcomes may help to clarify how systemic, ecological, and socio-cultural changes are perceived to contribute to wildness, resilience, and sustainability within the context of rewilding.

#### Wildness

Given the emphasis on "wild" within the term "rewilding," it comes as no surprise that wildness is a central aim, e.g. "a wilder Europe in the twenty-first century" (Helmer et al. 2015) and "a vision for a restored wild America" (Terborgh and Soule 1999b). There are some reflections on the meaning of wild, particularly around its origins in the German word for "will," describing places or creatures free from human domination or domestication (Snyder 1990; Johns 2019). The RPS data reflect a shift away from wilderness as a goal of rewilding, reflecting challenges to the concept of the wilderness ideal in the IRT (e.g. Boitani and Linnell 2015; Gammon 2019; Ward 2019). Wildness is explored as a subjective and multivalent term with meaning beyond non-human agency, including socio-cultural aspects and with the potential to occur outside of wilderness areas, among humans, or within humans (e.g. Snyder 1990; Prior and Ward 2016; Durant et al. 2019). Thus, wild can be considered a landscape-scale or SES aim, combining ecological and socio-cultural aims and outcomes highlighted in the remaining sections.

#### Resilience and sustainability

There are numerous considerations in the RPS and IRT for how rewilding promotes SES resilience and sustainability, in particular reflecting the desire for systems to become self-sustaining and with natural disturbance-recovery regimes. For example,

“The goal of rewilding is not to restore a painting that then needs curating, it is about restoring a system that can come to look after itself.” (RPS)

The data show a shift from purely ecological notions of resilience, considering the socio-cultural change necessary to achieve these states and the interdependence between human and ecological factors in SES. While specific studies of rewilding’s contribution to sustainability and resilience in the IRT tend to focus on ecological health or human health separately (as reflected in the following sections), the broader considerations for resilience and sustainability reflect mutual flourishing of human and non-human nature, e.g. "a better future for all" (Terborgh and Soule 1999b). Sustainability and resilience are also linked to concern that the outcomes of rewilding should persist, especially in the context of climate change (e.g. Barlow 1999; Carver 2009). The aims and outcomes presented in this paper may offer insights or potential leverage points for achieving system sustainability, a critical element of sustainability science (Abson et al. 2017).

#### Coexistence

Across the data, coexistence is seen as vital to the success of rewilding in theory and practice (e.g. Prior and Ward 2016; Durant et al. 2019) and is an outcome supported by the ecological and socio-cultural aims highlighted below. The concept of coexistence originated based on concerns over human–predator conflict (Linnell and Jackson 2019), but data show a shift towards conceptualisations of coexistence as integrated landscapes including humans, non-human species, and natural processes (e.g. Durant et al. 2019). These include human tolerance for ecological processes such as predation, death, decay, and disturbance (Groom et al., 1999; Noss et al., 1999; Linnell and Jackson 2019). The expansion of the concept to include integrated landscapes and tolerance for biotic and abiotic elements of SES reflects the shifting focus towards landscape or continental scale (Soule and Terborgh 1999a) and the need to consider the existence of different land uses, species and processes at scale. Given the complexity of SES, coexistence as a social–ecological quality requires further research within system science and in different contexts.

#### Reciprocity and cooperation

Reciprocity stems from a key driver of rewilding noted by RPS participants, a desire to “right the wrongs” associated with the Anthropocene and a need to address the imbalance of exploitation of natural resources. RPS participants cite the influence of Robin Wall Kimmerer who promotes reciprocal restoration, rooted in a balance of give-and-take (Wall Kimmerer 2013). Another key influence noted in the RPS is Leopold’s Land Ethic (Leopold 1949) that seeks to shift conservation towards an ethic of more-than-human cooperation. Landscape or SES aims of rewilding are therefore seen as being supported by notions of reciprocity and cooperation. This framing of rewilding may offer a route to alleviating the perceived paradox between non-human autonomy and interventions, where rewilding interventions are acts of cooperation or reciprocity. This is considered in a revised rewilding continuum in the concluding section.

#### System complexity and heterogeneity

The desired outcomes of landscape or SES complexity and heterogeneity are demonstrated in rewilding projects in the data. For example, Comins (2006) writes that the Tweed Rivers Heritage Project,

“Recognises that the landscape is a function of natural, social, and economic history and in managing this ‘tapestry’ it is necessary to look at all the threads that make it up.”

Taking a holistic approach to landscape planning was a motivation for the Wildlands Network in North America (Soule and Terborgh 1999a; Foreman 2004). Their 3Cs (cores, carnivores, corridors) approach to rewilding sought to designate core or protected areas where other-than-human nature dominates, and corridors between these areas allowing for a mosaic of land uses. Similar approaches to promote landscape heterogeneity are reflected elsewhere in the IRT (e.g. Vera 2000; Helmer et al. 2015; Corlett 2019). Acknowledging system heterogeneity and complexity allows for a broad spectrum of land uses, dependent on local conditions and inhabitants, integrating the needs of autonomous nature (which may include spatial and temporal requirements, diet, and habitat) together with the needs of humans (which may include governance, food, housing, recreation, and other needs related to culture and values). Integrating complexity and complex systems science into rewilding, landscape planning, policy, and decision-making presents a significant challenge. It requires advancements in system science application and the removal of sociopolitical barriers to understanding and integrating complexity and considering multiple actors (human and non-human) with diverse needs and values (Berkes et al. 2002).

### Ecological change

The role of ecological restoration in rewilding is prominent throughout the RPS data, with many participants equating rewilding with functional ecological restoration, demonstrating that rewilding characterizes a paradigm shift from compositional to functional restoration goals based on concerns with compositional conservation approaches (Carver et al. 2021). This emphasis reflects the considerable influence of ecologists on rewilding theory. However, it is also important to understand that ecological restoration in rewilding has specific ecological aims and outcomes outlined below. These are shared across the data, although there is some divergence over the value of indigeneity and ecosystems services, as highlighted below.

#### Non-human autonomy

As per Prior and Ward (2016), non-human autonomy is recognized as a pivotal ecological goal of rewilding, and across the RPS and IRT data there are links drawn between rewilding and the concept of wild or self-willed nature (e.g. Johns 2019). The concept encompasses two interrelated aspects: self-willed nature—acknowledging the autonomy of non-human nature or “*‘natura naturans’* (nature doing what nature does)” (RPS data)—and ecological resilience—recognizing nature’s ability to self-regulate or “look after itself” (RPS data). These aspects are driven by concerns over increasing human dominance and are often intertwined in the data, e.g. Foreman et al. (1992) consider “the requirements of all native species to flourish within the ebb and flow of ecological processes, rather than within the constraints of what industrial civilization is content to leave alone”. However, the idea of non-human autonomy is not without uncertainties, primarily linked to perceptions that it conflicts with human intervention or presence, as mentioned in Sect. "Introduction". The ecological outcomes outlined below may support more targeted questions, such as at what point or in what circumstances does human influence become incompatible with wildness or negatively influence ecological resilience.

#### Ecological integrity

To support the key aim of non-human autonomy, "ecological integrity" is considered in several IRT, emphasizing a holistic perspective that includes structure and function (e.g. Simberloff et al. 1999; Foreman 2004; Cortes-Avizanda et al. 2016). The intent to return missing components of degraded ecosystems is prevalent in the RPS, especially the reintroduction of species to re-instate their ecological roles. Ecological integrity places emphasis on the functioning of the whole system in relation to its parts or individual processes. This demonstrates that, although rewilding is primarily concerned with functional restoration, composition can remain influential, given that structure influences function, so reference ecosystems remain important. In practice, prioritization of functional restoration is likely to occur based on factors such as project resources, objectives, and the potential for certain elements to have a broader impact (Hawkins et al. 2024).

#### Ecological diversity and heterogeneity

Throughout the RPS are demonstrated value for biodiversity and ecological heterogeneity, highlighting “the connection between a healthy and intact landscape and the conservation of biological diversity” (RPS), linking this outcome with ecological resilience, ecological integrity, and other rewilding aims and outcomes. This emphasis is rooted in rewilding’s connections to conservation biology (e.g. Noss 1992; Soule and Noss 1998), the appeal of biodiversity to the public, and the potential for rewilding to contribute to global or local biodiversity or habitat objectives (e.g. Navarro and Pereira 2015).

#### Evolutionary potential

The restoration and conservation of evolutionary potential is considered by some as a significant outcome of rewilding, highlighting its contribution to long-term adaptation to change (e.g. Foreman et al. 1992; Barlow 1999; Donlan et al. 2005). This theme also recognizes the co-evolution of species within a system, stemming from the theory of ecological anachronisms (Janzen and Martin 1982; Barlow 2000), which is cited in the RPS as influential on conceptualizations of rewilding, particularly Pleistocene rewilding. However, there’s acknowledgement of the spatial and temporal requirements of evolutionary processes, highlighting the challenges of achieving evolutionary potential in modern society amid increasing human influence (Martin and Burney 1999; Barlow 2000; Vera 2000; Svenning et al. 2016).

#### Indigeneity

Words such as “native”, “original”, and “indigenous” are used in the RPS data, along with highlighting the need to return “missing species”, demonstrating the value that some place on indigeneity. It is seen to contribute to other rewilding aims, e.g. it is perceived as relevant to ecological integrity and resilience due to the co-evolution of biota (Leopold 1949; Barlow 2000) and therefore guides decisions on ecological composition during restoration or rewilding. However, there is variation on the value placed on indigeneity, reflecting support for the novel ecosystem concept in rewilding (e.g. Klop-Toker et al. 2020) as well as the use of non-native species as ecological surrogates and the influence of Pleistocene rewilding (Donlan et al. 2005; du Toit 2019). Thus, the value of indigeneity can be considered as relative to other ecological outcomes.

#### Provision of ecosystem services

The intent to improve ecosystem services through rewilding is reflected in IRT that emphasize the significance of rewilding in providing ecosystem services (e.g. Cerqueira et al*.*, 2015; Navarro and Pereira 2015; Pettorelli et al. 2018). Ecosystem services are thought to underpin SES resilience and sustainability, particularly in response to climate change. While some argue that enhancing ecosystem services is an explicit rewilding goal (Cerqueira et al*.*, 2015; Pettorelli et al. 2018), the IRT also reflect concerns around the ecosystem services concept (e.g. Taylor 2009), echoing similar concerns in the wider literature (Chan and Satterfield 2020). This is further considered under the section “Valuing nature”.

#### Connectivity

Connectivity is seen as integral to rewilding as it supports natural dispersal and associated ecological functions, particularly reflecting the large-scale emphasis of rewilding and the requirements of wide-ranging species. For example, connectivity is a focus of landscape mapping to inform rewilding (Soule and Terborgh 1999a; Carver 2022), improved connectivity is discussed as a potential outcome of trophic rewilding (Svenning et al. 2016; Bakker and Svenning 2018), or as enabling the return of large carnivores (von Arx and Breitenmoser 2004; Linnell and Jackson 2019). This aspect of rewilding is closely associated with coexistence and the cohabitation of humans and other species in landscapes.

### Socio-cultural change

The socio-cultural aims of rewilding are less explicitly discussed in rewilding literature than the ecological aims, but they are clearly understood by many participants of the RPS and authors of IRT as being crucial to rewilding, with an understanding that, to achieve ecological or landscape-scale aims, rewilding requires, and should therefore incorporate, socio-cultural change. Aims and outcomes reflect the intention for rewilding to promote socio-cultural change at different scales, from societal to individual level, reflecting engagement with rewilding from personal to systems levels, as reflected in interventions to support personal rewilding or cultural or policy change (Hawkins et al. 2024).

#### People accommodating wild nature in our landscapes

This aim is pivotal in realizing the ecological goal of non-human autonomy and implies the "letting", "allowing", and "giving" that is expressed throughout the RPS data, e.g. “giving more space to nature” or “allowing [natural processes] to shape the landscape”. The RPS data show that concerns for human dominance and control have influenced this aim, e.g. “Our biggest obstacle is the inability to take a hands-off approach to anything, we always want to manipulate and manage” (RPS). As a result, rewilding is considered to include some degree of reducing human control and management to accommodate the spatial and temporal requirements of natural processes. The term "our" in this context refers to the more-than-human community. “Accommodating” is a broad term which allows for varying contexts and interpretations, including doing nothing such as in passive rewilding or land abandonment (e.g. McKibben 1995; Carver 2019); tolerating or accepting the presence or return of wild nature (e.g. Bennett 2006; Linnell and Jackson 2019); actively withdrawing human influence and/or restoring wild nature which is associated with active rewilding interventions (Hawkins et al. 2024); or embedding wild nature into cultures and landscapes (e.g. Helmer et al. 2015; Prior and Ward 2016). It also aligns with emerging policies such as the ambition for “30 × 30” in the post 2020 global biodiversity framework (UNDP et al., 2021).

#### Human well-being

Across the RPS data is highlighted the potential for rewilding to have, e.g. “direct benefits to human health and well-being”, and several IRT explore the potential for rewilding to contribute to improving human well-being (McKibben 1995; Comins 2006; Sandom et al. 2013; Maller et al. 2019), such as the suggestion that wild nature “enriches our lives and nourishes our psyches” (Soule and Terborgh 1999b). Given the subjective nature of well-being (González et al. 2021), these sources consider different ways that rewilding might improve well-being, including the provision of ecosystem services, providing opportunities to diversify income and livelihood opportunities (particularly in rural communities), increasing opportunities for recreation, wildlife experiences, and outdoor education, and the related impacts on human–nature connection. However, there are also concerns that rewilding can negatively impact well-being, including livelihoods and sense of place, and this influenced those promoting rewilding to highlight rewilding’s benefits to humans (Prior and Ward 2016; Jepson et al. 2018). This has led to some tensions noted in the RPS around the degree to which rewilding should seek to directly benefit people, with concerns over anthropocentrism. However, this study demonstrates that rewilding aims holistically to balance social–ecological aims and therefore well-being is seen as relative to other aims and outcomes, whereby, for example, enhancing people’s tolerance for risk and unpredictability (noted as an outcome below) can influence people’s perceptions of well-being. This aligns rewilding with transformative goals, shifting people’s underlying values to enhance resilience (Fougères et al. 2022), considered further below. This further demonstrates the need for place-based approaches (Hawkins et al. 2024). How rewilding impacts well-being in various contexts and balances transformation with pragmatism (Hawkins et al. 2024) is a topic for further research.

#### Human–nature (re)connection and ecocentrism

Across the data, changes to human–nature relationships are emphasized as necessary for supporting rewilding aims, aligning with rewilding principles (Carver et al. 2021), the relational qualities of SES, and a broader relational turn in environmental literature (Eyster et al. 2023). Alan Watson Featherstone (2004) links rewilding with “healing the relationship between humanity and the rest of Nature”, while the RPS calls for “moving away from the current estrangement/alienation” of people from nature. This is related to relational values, discussed further in “valuing nature”. However, cultural differences in perceptions of nature and the conflict between dualistic and holistic ontologies (Sect. "Introduction") create uncertainty around the qualities of the intended human–nature relationship, leading this outcome to focus on (re)connection.

In response to concerns about anthropocentrism, many rewilding advocates promote ecocentrism (Carver et al. 2021; Rawles 2022). Ecocentrism, as defined here, is a holistic ontology that places humans within an interdependent system, prioritizing the health of the whole over species-specific well-being (Washington et al. 2017; Taylor et al. 2020), echoing Leopold’s (1949) Land Ethic. However, critics argue that ecocentrism is tied to wilderness concepts, complicating its application in cultural landscapes (Jepson et al. 2018).

Clayton (2019) introduces a scale of environmental identity (EID), where high EID reflects a strong connection to nature, varying from separation (low EID) to intimacy (high EID). A higher EID may correlate with people’s ability to coexist with non-human autonomy. Despite these insights, conflicts and uncertainties persist regarding ecocentrism, the nature of the human–nature relationship in rewilding, and the role of plural values in achieving rewilding goals.

#### Tolerance and adaptability

Tolerance and adaptability (for risk, uncertainty and unpredictability associated with wildness and natural dynamism) are linked with coexistence and the socio-cultural aims above (e.g. Taylor 2009; Monbiot, 2013; Boitani and Linnell 2015; Maller et al. 2019). These sources explore links between tolerance and nature perceptions, acknowledging that perceptions can influence the understanding of real versus perceived risks, linking this to ecological knowledge and human–nature connection.

#### Valuing nature

The theme of valuing nature focuses on human appreciation for nature and its potential to contribute to other rewilding goals and enhance acceptance of rewilding initiatives. Three types of values—intrinsic, relational, and instrumental—are highlighted extensively in the data, reflecting studies on the influence of these values on decision-making in the wider environmental literature (Chan et al. 2018). There is some divergence in the emphases on instrumental and intrinsic value. Intrinsic value is mainly emphasized by RPS participants from North America, noting the influence of deep ecology on their perceptions of rewilding and the need to avoid “mechanistic metaphors that devalue other lifeforms” (RPS data), while pragmatism is associated with European rewilding and the use of economic incentives or ecosystem services to promote the instrumental value of non-human nature to people (Jepson et al. 2018; Pettorelli et al., 2018).

Given that rewilding application is place-based (Carver et al. 2021; Hawkins et al. 2024), these different types of value could have varied influence based on the diversity of stakeholders, and so there is the potential for value pluralism to be linked to system complexity and diversity recognizing multiple values across complex systems. The need for value pluralism to mitigate conflicts in environmental decision-making is gaining support in wider academic literature (Carmenta et al. 2023).

#### Ability to identify and prevent unsustainable activities

The RPS data highlight extensively the need for some reduction in unsustainable human activities, and so this outcome is seen to support this shift. This involves understanding the limitations of human influence or alterations within an ecological system, including the need to restrict overexploitation and other human-induced forms of harm. Leopold (1949) explores various aspects of human influence on ecological processes and the fine line between use and misuse, while Wall Kimmerer (2013) notes links between indigenous knowledge and sustainable harvesting, or “the honourable harvest”. These references and other IRT (e.g. Snyder 1990; Bauer and von Atzigen 2019; Linnell and Jackson 2019) link this outcome to ecological knowledge, valuing nature, monitoring, as well as some form of governance or enforcement. Place-based studies will enhance understanding of this outcome and its links to rewilding.

#### Connection to place

This outcome demonstrates the intention, reflected in the IRT, to reconnect people and their cultural identity to place (e.g. Snyder 1990; Vera 2000; Helmer et al. 2015). "Place" provides the setting for rewilding, emphasizing the importance of place-based approaches in rewilding application in the projects highlighted in the RPS data. Snyder (1990) notes that place connection not only supports wildness but also fosters cultural diversity, with links noted to bioregionalism. However, tensions are also noted, where place identity can act as a barrier to rewilding (Monbiot 2013), and so fostering change to place identity is seen as part of the rewilding process in degraded areas, particularly those influenced by shifting baseline syndrome (RPS data). This is reflected in the development of an "ethic of place" for rewilding, which critically analyses and deepens the sense of place (Drenthen 2022).

#### Ecological knowledge

Ecological knowledge is a fundamental component highlighted in several of the other socio-cultural outcomes above, making it crucial to acknowledge separately. This quality emphasizes the need to enhance people’s ecological knowledge, demonstrated by various ecological studies and theories influencing rewilding practice. Knowledge is closely associated with improving people’s understanding of the needs of other-than-human nature with the potential to influence tolerance of wildness. It also enhances the understanding of the health of a landscape and is associated with sustainable resource use. In an example from the IRT data, Monbiot notes that his willingness to accommodate wild nature was supported by knowledge:

“Only when I lived among ecosystems which retained many of their trophic levels, their diversity and dynamism, did I begin to understand how the natural world might work.”

These links are reflected in the wider literature, noting positive correlations between coexistence, ecological knowledge, and tolerance (Richardson et al. 2022; Lambert and Berger; 2022; Beery et al. 2023).

## Discussion and conclusion

In this paper, we provide a comprehensive examination of rewilding aims from a broad study of influences on the rewilding concept (namely influential people and texts), adopting a transdisciplinary approach that recognizes the necessity for social–ecological, ecological, and socio-cultural change. Aims and outcomes for these three dimensions are synthesized (Fig. 1), providing a holistic picture of the social–ecological aims and outcomes of rewilding that have influenced emergent rewilding theories and practice. The study highlights the intention for rewilding to affect transformative or systemic change, echoing previous considerations for its transformative capacity (Durant et al. 2019; Hawkins et al. 2022).

**Fig. 2:**
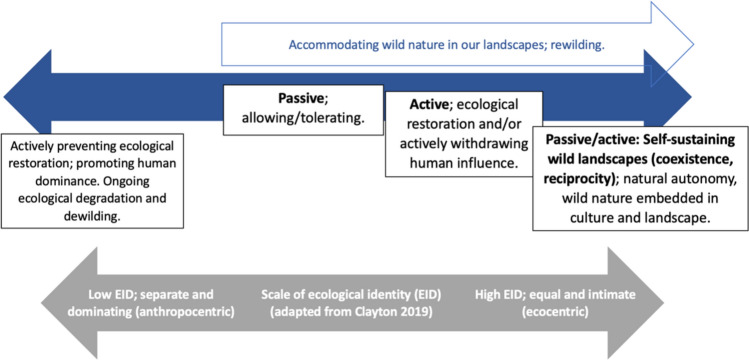
Continuum of people accommodating wild nature in landscapes, demonstrating how this aim contributes to rewilding at a landscape scale and connects to concepts such as protection, restoration, and stewardship. Also integrating the scale of EID, adapted from Clayton (2019)

The study supports the creation of a redefined rewilding continuum focusing on the socio-cultural aim of people accommodating wild nature in our landscapes (Fig. 2). This continuum demonstrates the spectrum of accommodating nature, ranging from passive to active, and from active transitioning to embedded and self-sustaining. This revised continuum addresses a perceived contradiction between active rewilding and non-human autonomy as reflected in previous rewilding frameworks (e.g. Torres et al., 2018; Perino et al. 2019). Additionally, it highlights opposition to accommodating nature, reflecting barriers to rewilding, often rooted in policies, individuals, or values that perpetuate unsustainable activities. This approach supports a rewilding vision of integrated landscapes or SES and a passive/active state maintained through ongoing adaptation to change and risk. This demonstrates links between dualistic ontologies—including anthropocentrism (Cocks and Simpson, 2015; Kopnina et al., 2018)—and ongoing ecological degradation, and links between positive EID/holistic ontologies and tolerance for wild nature. This latter link has been considered in the wider literature to support sustainability and resilience of SES (Cocks and Simpson, 2015; Washington et al. 2017). Significantly, the framework incorporates aims of coexistence, non-human autonomy, and people accommodating nature in our landscapes at a landscape scale and shows that these can be supported by active intervention (Hawkins et al. 2024), addressing perceived conflicts between intervention and non-human autonomy.

While the emergent theories from this study (depicted in Figs. 1 and 2) are simplified, they serve as foundational frameworks that integrate the breadth of perspectives on rewilding. This is an important step towards clarity and consensus in rewilding and can act as a basis to inform future research and development of tools for planning and monitoring, with the outcomes identified offering measurable indicators. The study also contributes to wider research on coexistence; human–nature relationships; and landscape or SES change or transformation. Given the breadth of the study, more focused research particularly drawing on different social–ecological contexts would be of value.

Despite its limitations, the presented aims offer a valuable evidence-based foundation for developing rewilding practice, frameworks, and theories, offering a focal point for identifying areas requiring further research or refinement. It also offers a rewilding vision, adaptable to different contexts, to encourage the rewilding community to collaborate towards common goals, explore the interconnections between ecological and social systems, and share experiences and lessons to enhance rewilding’s potential across systems, cultures, and disciplines.

## Supplementary Information

Below is the link to the electronic supplementary material.

Supplementary file1 (PDF 239 KB)

## Data Availability

The survey data used in this study are not anonymous and the respondents have not given permission to share the data in a repository. Please contact the author for requests.
