# Supplementary material for: Rewilding’s social–ecological aims: Integrating coexistence into a rewilding continuum
Source: Ambio. 2024 Dec 31;54(5):869–81. doi: 10.1007/s13280-024-02118-0 (PMC11965037; doi:10.1007/s13280-024-02118-0)
Supplement: Supplementary file 1 — Supplementary file1 (PDF 239 KB) [file 13280_2024_2118_MOESM1_ESM.pdf]

Ambio

Supplementary Information
This supplementary information has not been peer reviewed.

Title: Rewilding’s social-ecological aims: Integrating coexistence into a rewilding continuum

RPS survey questions

Introduction and consent

1. By ticking ‘I agree’ you consent that you have understood the information provided about this study, and wish to take part in the research.
   O I agree

We will now ask you 19 questions about Rewilding “then”, Rewilding “now” and Rewilding in practice. This will be followed by some standard demographic questions.

Rewilding “then”

These questions relate to the period when you first became aware of ‘rewilding’.

2. When, where and how did you first hear about rewilding (please include a year if possible)?
3. At that time, what did you understand rewilding to mean?
4. Who were the influential people/organisations involved in rewilding at that time?
5. At that time, what were your expectations for the field of rewilding?
6. What in your opinion were the circumstances/drivers at the time that you think gave rise to the concept of rewilding?
7. Back then, what were the most significant barriers to rewilding?
8. In your opinion, who are the rewilding pioneers we should be contacting?

Rewilding “now”

The questions on this page relate to your current understanding of rewilding and opinions on the future of the field.

9. Thinking about now – what do you understand rewilding to mean?
10. Do you have a preferred definition of rewilding, e.g. one from academic or practitioner literature?
11. Do you think rewilding has lived up to your expectations (as listed in question 5)?
12. Who would you consider to be the influential people/organisations involved in rewilding now?
13. What would you consider to be the most significant contemporary barriers to rewilding?
14. Please list any words or phrases you know, from any language, that you feel are synonymous with rewilding?
15. Do you think there are language and/or cultural barriers with the term ‘rewilding’? Please elaborate.
16. The term rewilding generates a wide range of opinion in popular media. The list below has been drawn from various media sources – which of these words would you associate with rewilding? (tick all that apply)
    - Bold
    - Innovative
    - Misanthropic
    - Flawed
    - Contradictory
    - Exciting

- Promising
- Controversial
- Ambitious
- Powerful
- Threatening
- Destructive
- Other (please specify):

17. To what extent would you agree with the following statement: *Rewilding is a positive term within the 'conservation community'*
    - Strongly agree
    - Agree
    - Neither agree nor disagree
    - Disagree
    - Strongly disagree
    - Other (please specify):
18. What do you see in the future of rewilding?

Rewilding in practice

These questions relate to the practical application of rewilding, if you have not been involved with any such projects then you can skip this page.

19. In which country/countries have you worked (or continue to work) on rewilding projects?
20. How has the concept of rewilding been received in communities you have worked with?

Just a few questions about you…

21. Please give your name.
22. What is your normal country of residence?
23. What is your gender?
    - Female
    - Male
    - Other (specify)
24. Please input your affiliation(s) in the textbox below.
25. What is your age?
    - 17 or younger
    - 18-20
    - 21-29
    - 30-39
    - 40-49
    - 50-59
    - 60 or older
26. If you would like to receive email updates from the IUCN Rewilding Task Force, please add your preferred email address below.

Supplementary Table 1. The list of IRT analysed in this study. These were all cited in the RPS data as influential on rewilding conceptualisations or practice. Although some texts are not about rewilding, these offer insights into how rewilding has been influenced, but conclusions about rewilding practice were not drawn directly from these texts.

**Year of publication**	**Authors/editors**	**Title**	**Type**	**Source**
1862	Thoreau, Henry David	Walking	Essay/speech	NA
1948	Leopold, Aldo	The Land Ethic	Book chapter	A Sand County Almanac
1982	Janzen, D.H. and Martin, P.S.	Neotropical Anachronisms: The Fruits the Gomphotheres Ate	Journal article	Science
1987	Newmark, W.D.	A Land-bridge Island Perspective on Mammalian Extinctions in Western North-American Parks	Journal article	Nature
1990	Snyder, Gary	Practice of the Wild	Book (monograph)	NA
1992	Foreman, Dave	Around the campfire	Magazine article	Wild Earth
1992	Foreman, Dave *et al.*	The Wildlands Project Mission Statement	Magazine article	Wild Earth
1992	Noss, Reed	The Wildlands Project: Land Conservation Strategy	Magazine article	Wild Earth
1995	Newmark, W.D.	Extinction of Mammal Populations in Western North American National Parks	Journal article	Conservation Biology
1995	McKibben, Bill	An explosion of green	Magazine article	The Atlantic
1998	Soulé, M. and Noss, R.	Rewilding and Biodiversity: Complementary Goals for Conservation	Magazine article	Wild Earth
1999	Barlow, C.	Rewilding for Evolution	Magazine article	Wild Earth

1999	Martin, P. and Burney, D.	Bring back the elephants	Magazine article	Wild Earth
1999	Soulé, M. and Terborgh, J. (eds)	Continental Conservation: Scientific Foundations of Regional Reserve Networks	Book (edited)	NA
2000	Barlow, Connie	The Ghosts of Evolution: Nonsensical Fruit, Missing Partners, and Other Ecological Anachronisms	Book (monograph)	NA
2000	Vera, Frans	Grazing Ecology and Forest History	Book (monograph)	NA
2004	Foreman, Dave	Rewilding North America	Book (monograph)	NA
2005	Donlan, J. *et al.*	Re-wilding North America	Journal article	Nature
2011	Taylor, Peter (ed)	Rewilding: ECOS writing on wildland and conservation values	Book (edited)	Chapters originally published in ECOS
2013	Monbiot, George	Feral	Book (monograph)	NA
2013	Wall Kimmerer, Robin	Braiding Sweetgrass	Book (monograph)	NA
2015	Pereira, H.M. and Navarro, L.M. (eds)	Rewilding European Landscapes	Book (edited)	NA
2016	Prior, J. and Ward, K.	Rethinking rewilding: A response to Jorgensen	Journal article	Geoforum
2016	Svenning, J.C. *et al.*	Science for a wilder Anthropocene: Synthesis and future directions for trophic rewilding research	Journal article	PNAS

2016	Jepson, P. and Schepers, F.	Making space for rewilding: Creating an enabling policy environment	Policy brief	Rewilding Europe
2018	Jepson, P., Schepers, F., and Helmer, W.	Governing with nature: a European perspective on putting rewilding principles into practice	Journal article	Philosophical Transactions of the Royal Society B
2018	Gammon, Andrea	The Many Meanings of Rewilding: An Introduction and the Case for a Broad Conceptualisation	Journal article	Environmental Values
2018	Pettorelli, N. *et al.*	Making rewilding fit for policy	Journal article	Journal of Applied Ecology
2018	Bakker, E. and Svenning, J.C.	Trophic rewilding: impact on ecosystems under global change	Journal article	Philosophical Transactions of the Royal Society B
2019	Pettorelli, N., Durant, S., and du Toit, J. (eds)	Rewilding	Book (edited)	NA
